# Insanity

**Published:** 1884-06

**Authors:** 


### EDITORIAL NOTES.

INSANITY.—A number of months since an editorial on Insanity appeared in this journal. The views expressed, we have reason to believe, were somewhat heterodox. An assistant physician on the staff of a State insane asylum, in commenting upon the argument, remarked that it contained some truth mixed with a great deal of error. Before accepting this rather dogmatic opinion, we invite the physician in question, or some other member of the profession, to prove his assertion.

ATTENTION has recently been called to the value of oil of wintergreen in acute rheumatism. As it is mainly a methyl salicylate, it is reasonable to expect better results from it than from other salicylates of a more fixed character. The objection to its use has been the difficulty of administration. This objection is entirely overcome by the use of the oil in capsules. The well-known and reliable house of Planten & Son now prepare them in two sizes, five and ten minims each.

DR. PARK RITCHIE, of St. Paul, states: "Am prescribing Tongaline with satisfactory results. For the indefinite aches and pains of nervous patients it is superior to any other anodyne. For nervous headache it is almost a specific." Dr. C. H. Hughes, of St. Louis, states: "Have found Tongaline a useful combination in rheumatic neuralgia." Dr. T. L. Bell, of Louisville, states: "Have used Tongaline in neuralgic affections, many of them of a severe form, with the most gratifying results."

Dr. J. K. Bauduy, Prof. of Nervous and Mental Diseases, Missouri Medical College, says: "After a thorough and continued trial of Bromidia at St. Vincent's Asylum, I can cheerfully certify to its great therapeutic value and purity. Its effects are much more rapid and efficient than the ordinary chloral mixtures. The sisters in charge of the wards, ofter using the Bromidia and comparing its effects with the ordinary chloral mixtures used so long as a hypnotic, claim great superiority for the former. Its success has been tested where the other, in similar doses, has failed. The purity of the chloral and the extracts of cannabis indica and the hyoscyamus which it contains, together with the small doses of the remedy which are required, make it almost invaluable to medical practitioners, who are guaranteed a pure and efficacious remedy in the use of Bromidia. They are not left at the mercy of pharmacists, who sometimes dispense inferior if not adulterated preparations of chloral. We could not, for some time, be induced to try the remedy, entertaining some prejudice against all such preparations. But experience in its use requires us, as a matter of justice, most emphatically to indorse the preparation after an extended and impartial trial. In fact, we expect in future to use Bromidia exclusively."

